# Determination of Substrate Preferences for Desaturases and Elongases for Production of Docosahexaenoic Acid from Oleic Acid in Engineered Canola

**DOI:** 10.1007/s11745-017-4235-4

**Published:** 2017-02-14

**Authors:** Jenny Lindberg Yilmaz, Ze Long Lim, Mirela Beganovic, Steven Breazeale, Carl Andre, Sten Stymne, Patricia Vrinten, Toralf Senger

**Affiliations:** 1 Scandinavian Biotechnology Research (ScanBiRes) AB, 230 53 Alnarp, Sweden; 2 Bioriginal Food and Science Corporation, Saskatoon, SK S7N 0W9 Canada; 3 BASF Plant Science LP, Research Triangle Park, NC 27709 USA; 4 Department of Plant Breeding, Swedish University of Agricultural Sciences, 230 53 Alnarp, Sweden

**Keywords:** Lipid biochemistry, n-3 Fatty acids, Nutrition, Elongases, Desaturases, Polyunsaturated fatty acids (PUFA), Genetic engineering, Phosphatidylcholine, Acyl-CoA

## Abstract

Production of eicosapentaenoic acid (EPA) and docosahexaenoic acid (DHA) in plant seed oils has been pursued to improve availability of these omega-3 fatty acids that provide important human health benefits. Canola (*Brassica napus*), through the introduction of 10 enzymes, can convert oleic acid (OLA) into EPA and ultimately DHA through a pathway consisting of two elongation and five desaturation steps. Herein we present an assessment of the substrate specificity of the seven desaturases and three elongases that were introduced into canola by expressing individual proteins in yeast. *In vivo* feeding experiments were conducted with 14 potential fatty acid intermediates in an OLA to DHA pathway to determine the fatty acid substrate profiles for each enzyme. Membrane fractions were prepared from yeast expression strains and shown to contain active enzymes. The elongases, as expected, extended acyl-CoA substrates in the presence of malonyl-CoA. To distinguish between enzymes that desaturate CoA- and phosphatidylcholine-linked fatty acid substrates, we developed a novel *in vitro* method. We show that a delta-12 desaturase from *Phytophthora sojae*, an omega-3 desaturase from *Phytophthora infestans* and a delta-4 desaturase from *Thraustochytrium* sp., all prefer phosphatidylcholine-linked acyl substrates with comparatively low use of acyl-CoA substrates. To further validate our method, a delta-9 desaturase from *Saccharomyces cerevisiae* was confirmed to use acyl-CoA as substrate, but could not use phosphatidylcholine-linked substrates. The results and the assay methods presented herein will be useful in efforts to improve modeling of fatty acid metabolism and production of EPA and DHA in plants.

**Electronic supplementary material:**

The online version of this article (doi:10.1007/s11745-017-4235-4) contains supplementary material, which is available to authorized users.

## Introduction

Plant oils have versatile use in both food and non-food sectors. The fatty acid composition governs the physical, chemical and nutritional properties of the oil, and while largely genetically controlled, is also subject to environmental regulation. Natural selection, mutation breeding, genetic engineering and, recently, site-directed mutagenesis have been used to modify the proportion of the existing fatty acid in the oil in many oil crops. Such examples include low erucic rape seed (Canola) and high oleic sunflower, canola and soybean [1–5]. Genetic engineering has also been used to introduce several novel fatty acids for both non-food and food usage into transgenic oil seeds [6].

The health benefits of the very long chain, omega-3 fatty acids, eicosapentaenoic (EPA, 20:5n-3) and docosahexaenoic acid (DHA, 22:6n-3) are well recognized [7] and have led various global health organizations to issue recommendations for dietary intake of these fatty acids [8]. Marine fish, the main nutritional source for EPA and DHA, have a very limited ability to produce these fatty acids. Rather, the fatty acids are synthesized by microorganisms and then accumulated up the food chain into the fish and other marine organisms that are used for human consumption. These omega-3 fatty acids are also important ingredients in feed utilized for commercial marine aquaculture. In some microorganisms, EPA and DHA are made through an anaerobic polyketide pathway involving mega-synthases (i.e. PUFA synthases) [9, 10]. More commonly however, EPA and DHA are synthesized aerobically from oleic (OLA, 18:1n-9), linoleic (LNA, 18:2n-6) and linolenic acid (18:3n-3 and 18:3n-6), fatty acids that are also common in oil crops, through a pathway employing discrete elongase and desaturase enzymes [11].

In an effort to provide an alternative, cost-effective and, renewable source of omega-3 fatty acids, considerable interest has been devoted to engineer oil seeds to produce EPA and DHA [12–15]. Aerobic conversion of OLA into DHA requires five desaturation and two elongation steps. The exact biosynthetic route from OLA to DHA is largely governed by the fatty acid and acyl-backbone specificities of the individual enzymes [16, 17]. Regardless of the route of synthesis, a bottleneck in the production of EPA and DHA in plants has been assumed to be the movement of acyl groups between the phosphatidylcholine pool for desaturation and the acyl-CoA pool for elongation [16]. However, this hypothesis has not been substantiated by biochemical studies regarding the acyl carrier to which the acyl group is linked in the different desaturation steps to DHA synthesis and conclusive evidence can only be obtained by *in vitro* studies of the enzymes.

**Fig. 1:**
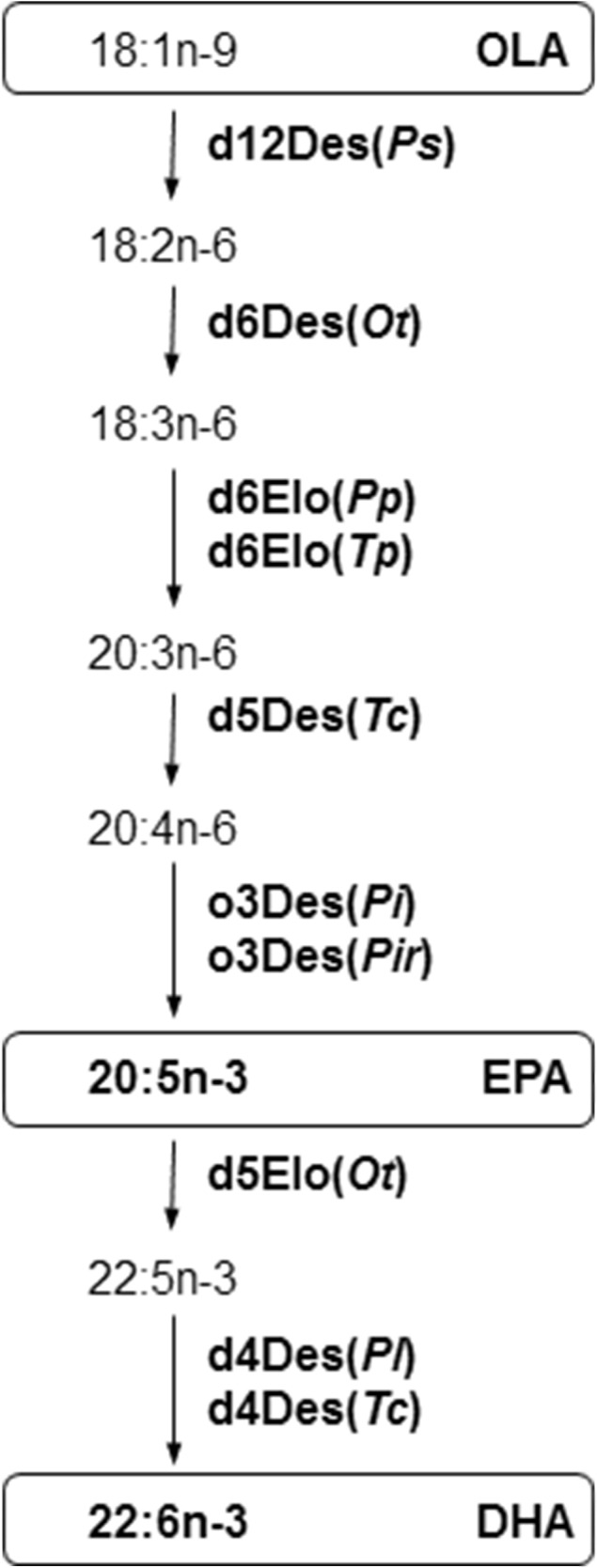
A pathway involving two elongation steps and five desaturation steps allows production of EPA (20:5n-3) and DHA (22:6n-3) from oleic acid (OLA, 18:1n-9). In this pathway seven desaturase enzymes (Des) and three elongase enzymes (Elo), further described in Table 1, modify fatty acid substrates that are covalently bound to a carrier (e.g. coenzyme A or phosphatidylcholine). Shown is a simplified route for synthesis of DHA that only describes a fatty acid moiety, but not the acyl-carrier, that is recognized by each enzyme

Shown in Fig. 1 and described in Table 1 are the seven desaturases and three elongases that were introduced into canola (*Brassica napus*) to confer production of DHA and its biosynthetic intermediate EPA from endogenous fatty acids [18]. Using yeast strains that expressed individual proteins we provide an assessment of the substrate tolerance for each of the seven desaturases and three elongases that were introduced into canola. We determined fatty acid substrate profiles for each of these enzymes using *in vivo* feeding studies for 14 different fatty acids that could be generated during DHA biosynthesis. We also show the *in vitro* activity for the individual desaturases and elongases expressed in yeast. Notably, we developed assay methods to discriminate acyl-CoA linked desaturation from phosphatidylcholine-linked desaturation. The delta-9 desaturase (OLE1) from *S. cerevisiae* was confirmed to use an acyl-CoA substrate whereas three desaturases in the DHA synthesis pathway were shown to utilize substrates comprised of fatty acids covalently bound to phosphatidylcholine.

**Table 1:** Genes encoding desaturases and elongases involved in DHA biosynthesis

Gene name	Enzymatic function, abbreviation, and source	Genbank protein accession number
*d12Des(Ps)*	Delta-12 desaturase, d12Des(*Ps*), from *Phytophthora sojae*	EGZ11023
*d6Des(Ot)*	Delta-6 desaturase, d6Des(*Ot*), from *Ostreococcus tauri*	XP_003082578
*d6Elo(Pp)*	Delta-6 elongase, d6Elo(*Pp*), from *Physcomitrella patens*	AAL84174
*d6Elo(Tp)*	Delta-6 elongase, d6Elo(*Tp*), from *Thalassiosira pseudonana*	XP_002288481, with P196S substitution
*d5Des(Tc)*	Delta-5 desaturase, d5Des(*Tc*), from *Thraustochytrium* sp. ATCC21685	AAM09687
*o3Des(Pi)*	Omega-3-desaturase, o3Des(*Pi*), from *Phytophthora infestans*	XP_002902599
*o3Des(Pir)*	Omega-3 desaturase, o3Des(*Pir*), from *Pythium irregulare*	AME80860
*d5Elo(Ot)*	Delta-5 elongase, d5Elo(*Ot*), from *Ostreococcus tauri*	CAI58913
*d4Des(Pl)*	Delta-4 desaturase, d4Des(*Pl*), from *Pavlova lutheri*	AAQ98793
*d4Des(Tc)*	Delta-4 desaturase, d4Des(*Tc*), from *Thraustochytrium* sp.	CAX48933

## Materials and Methods

### Chemicals

Fatty acids and Coenzyme A were obtained from Nu-Chek Prep Inc (Elysian, MN) and from Larodan (Malmö, Sweden), [1-^14^C]Fatty acids from PerkinElmer or Lipidox (Stockholm, Sweden). Palmitoyl(16:0)-lysophosphatidylcholine (16:0-lysoPtdCho), essentially fatty acid free bovine serum albumin (BSA), and other fine chemicals were purchased from Sigma-Aldrich (St. Louis, USA). [^14^C]Acyl-CoA and acyl-CoAs were synthesized according to the method described by [19]. Argentation TLC plates were prepared by immersing TLC plates (silica 60, Merck) in AgNO_3_ (12%, w/v) dissolved acetone/water (4:1 by vol.) and drying the plates at room temperature.

### Yeast *In Vivo* Fatty Acid Feeding Studies: Gene Cloning

Protein coding genes (Table 1), codon-optimized for expression in *B. napus*, were synthesized (Geneart, Regensburg) and cloned into the yeast expression vector pYES2.1/V5-His-TOPO (Invitrogen) under the control of the Gal1 promoter. Each coding sequence contained a stop codon to prevent translation of C-terminal epitope tags. After confirming the sequences, plasmids were transformed into strain INVSc1 (Invitrogen), using the Sc EasyComp Transformation Kit (Invitrogen) and selected for the presence of the introduced plasmids on plates lacking the appropriate amino acids.

The fatty acid 20:4n-3 was not commercially available, and was generated *in vivo* by co-expression of d6Elo(*Tp*). For this purpose, the codon optimized *d6Elo(Tp)* gene was cloned into the yeast expression vector pESC-Leu under the control of the Gal1 promoter. Yeast strain INVSc1 (Invitrogen) was co-transformed with pESC-Leu containing *d6Elo(Tp)* and with pYES2.1/V5-His-TOPO containing other desaturase and elongase genes. Induction with galactose therefore resulted in the expression of d6Elo(*Tp*) and a second enzyme. Upon feeding with stearidonic acid (SDA, 18:4n-3), 20:4n-3 produced by d6Elo(*Tp*) was available as a substrate for the desaturase or elongase under investigation.

### Yeast *In Vivo* Fatty Acid Feeding Studies: Protein Expression and Fatty Acid Analysis

Yeast cultures were grown overnight at 30 °C in drop out base (DOB-URA) containing 2% glucose. The samples were then washed with DOB-URA containing 2% galactose and suspended in DOB-URA plus 2% galactose at a final OD_600_ = 0.66. Expression was carried out for 3 days at 20 °C in the same media, supplemented with 0.25 mM exogenous fatty acids and 0.01% tergitol.

For fatty acid analysis, 5 mL of culture volume was precipitated by centrifugation and washed once with induction buffer and once with water. The supernatant was removed and 2 mL of 3 N methanolic-HCl (Supelco) was added to the cell pellet. After gentle mixing, the mixture was incubated at 80 °C for 40 min, cooled to room temperature, and 1 mL 0.9% NaCl plus 2 mL hexane were added. After mixing, the hexane phase was removed and dried under nitrogen gas. Fatty acid methyl esters (FAME) were resuspended in 100 µl hexane and analyzed by gas chromatography using an Agilent 6890 N gas chromatograph equipped with a DB-23 column. The thermal program used was 160 °C for 1 min, then the temperature was increased to 240 °C at a rate of 4 °C/min. FAME were identified based on known standards and the conversion percentage was calculated as: [(product) × 100%]/(substrate + product). The analysis was repeated with at least three cultures originating from independent yeast colonies.

### Yeast *In Vitro* Enzyme Activity Assays: Gene Cloning

Protein coding genes (Table 1), codon-optimized for expression in *B. napus*, were synthesized and cloned into the yeast expression vector pYES2 under the control of the GAL1 promoter. N- or C-terminal 3xFLAG tags were incorporated into the coding sequence of each gene. An N-terminal tag was used for d12Des(*Ps*), d6Des(*Ot*), d6Elo(*Pp*), d6Elo(*Tp*), o3Des(*Pir*), and d4Des(*Pl*) proteins. A C-terminal tag was used for d5Des(*Tc*), o3Des(*Pi*), d5Elo(*Ot*), and d4Des(*Tc*) proteins. All sequence of each of these constructs was confirmed prior to yeast transformation. *S. cerevisiae* strain Sc334 [20] was used for all transformations.

The endogenous yeast gene *OLE1*, encoding a delta-9 desaturase [21], was amplified from genomic DNA from Sc334 using PCR. The amplified *OLE1* sequence was cloned into vector pJET1.2 by blunt-end ligation and the coding sequence was verified by DNA sequencing. The *OLE1* fragment was then excised from pJET1.2 and cloned into the yeast expression vector pYES2. The construct was transformed into the *S. cerevisiae* strain SCY62 [22].

### Yeast *In Vitro* Enzyme Activity Assays: Preparation of Membranes

Recombinant yeast cells were grown at 30 °C in DOB-URA containing 2% galactose. After 24 h, yeast cells were harvested, washed with 25 mM Tris–HCl, pH 7.6, and resuspended in Desaturase Disruption Buffer (0.1 M potassium phosphate pH 7.2, 0.33 M sucrose, 1 mg/ml BSA, 4 mM NADH, 4000 U/ml catalase) or Elongase Disruption Buffer (20 mM Tris–HCl, pH 7.9, 10 mM MgCl_2_, 1 mM EDTA, 5% (v/v) glycerol, 0.3 M ammonium sulfate) containing protease inhibitor (Complete, Roche Applied Science). The cells were disrupted by homogenization with 0.5-mm zirconia/silica beads using a Mini Beadbeater-8 (Biospec Products). The homogenates were centrifuged at 1500×*g*, and supernatants were transferred to new tubes, diluted with the appropriate disruption buffer, and centrifuged at 100,000×*g* for 2 h at 4 °C. The pellets were resuspended in Desaturase Disruption Buffer or Elongase Assay Buffer (50 mM HEPES–KOH pH 6.8, 150 mM potassium acetate, 2 mM magnesium acetate, 1 mM CaCl_2_) with protease inhibitor, and these membrane protein extracts, also referred to as membrane fractions or membranes, were used directly or stored at −80 °C.

### Yeast *In Vitro* Enzyme Activity Assays: Observing Total Acyl Modification

The elongase assays contained 200 µg membrane protein, 7.5 nmol [malonyl-2-^14^C]malonyl-CoA (3000 dpm/nmol), 5 nmol acyl-CoA (18:3n-6-CoA in the delta-6 elongase assays and 20:5n-3-CoA in the delta-5 elongase assays), 1 mM NADPH, 2 mM MgCl_2_, 100 µM cerulenin, and elongase assay buffer to a total volume of 100 µl. The assays were incubated 1 h at 30 °C.

The desaturase assays contained 100 µg of membrane protein, 10 nmol [^14^C]acyl-CoA (3000 dpm/nmol), 7.2 mM NADH, 1.8 mg/ml BSA in desaturase assay buffer (0.1 M potassium phosphate pH 7.2, 0.33 M sucrose, 4000 U/ml catalase, protease inhibitor) to a total volume of 200 µl. In some cases the assay contained 10 nmol of 16:0-lysoPtdCho, as indicated in the figure legends. The assays were incubated 1 h at 30 °C, unless indicated otherwise.

### Yeast *In Vitro* Enzyme Activity Assays: Observing Phosphatidylcholine-Linked Acyl Desaturation

To test if a desaturase accepts a phosphatidylcholine-linked substrate, the enzyme reaction was performed as described above for the desaturase assays (“observing total acyl modification”), except that the membrane fraction of the yeast strain expressing the enzyme of interest was pre-incubated with [^14^C]labeled acyl-CoA for 15 min in the presence of exogenous 50 µM 16:0-lysophosphatidyl choline (lysoPtdCho), before adding NADH. The membranes used in these assays were prepared without NADH in the disruption buffer but otherwise as described above. The reactions were allowed to continue for 0–120 min (at 30 °C) after NADH addition and then the lipids were extracted and analyzed as described under “lipid separation and analysis”.

### Yeast *In Vitro* Enzyme Activity Assays: Observing Coenzyme A-Linked Acyl Desaturation

The assay conditions were as described above for the desaturase assays (“observing total acyl modification”) with the following modifications: (1) the membrane fraction of the yeast strain expressing the enzyme of interest was prepared without NADH in the Desaturase Disruption Buffer and (2) a pre-incubation with 10 nmol 20:1n-9-CoA (50 µM) and 0.5 mM 5,5′-dithiobis-(2-nitrobenzoic acid) (DTNB) for 10 min was included before addition of NADH and [^14^C]labeled acyl-CoA substrate. The reactions were allowed to continue for 0–120 min (at 30 °C) after NADH addition and the lipids were extracted and analyzed as described under “lipid separation and analysis”.

### Yeast *In Vitro* Enzyme Activity Assays: Lipid Separation and Analysis

A solution of 100 µl of 2 M potassium hydroxide in methanol/water (1:4 v/v) was added to the elongase assay reactions followed by incubation for 20 min at 90 °C. Fatty acids were extracted according to Bligh and Dyer [23] by addition of 100 µl 3 M HCl, 750 µl methanol/chloroform (2:1 v/v) and then 250 µl chloroform. To the desaturase reactions described for “total acyl modification”, 200 µl of 2 M potassium hydroxide in methanol/water (1:4 v/v) was added and after incubation for 20 min at 90 °C, fatty acids were extracted by addition of 200 µl 3 M HCl, 1.5 ml MeOH:CHCl_3_ (2:1 v/v) and 500 µl CHCl_3_. The chloroform phases were recovered and dried under N_2_(g). Fatty acids were methylated by addition of 2 ml methanol containing 2% sulfuric acid at 90 °C for 30 min. Fatty acid methyl esters from the elongase assays were extracted by addition of 2 ml water and 2 ml n-hexane and separated by reverse-phase (RP) TLC (TLC Silica gel 60 RP-18 F254S, Merck) in acetonitrile/tetrahydrofuran (85:15 v/v). The methyl esters from the desaturase assays were separated by RP-TLC in 100% acetonitrile or by argentation TLC (heptane:diethylether:acetic acid, 70:30:1 v/v/v). Radioactive methyl esters were visualized and identified by Rf values of authentic standards in an Instant Imager (Packard Instrument Co.) electronic autoradiograph and quantified by scintillation counting (PHILIPS liquid scintillation counter system PW4700).

In the assays observing phosphatidylcholine- or CoA-linked acyl-desaturation, lipids were instead extracted after the reaction, by the addition of 200 µl 0.15 M acetic acid and 1 ml methanol/chloroform (1:1 v/v). The methanol/water-phase was washed twice with 0.5 ml chloroform and the chloroform fractions were pooled. An aliquot (about 10%) of the chloroform phase, containing phosphatidylcholine (PtdCho) and free fatty acids, was measured by scintillation counting and the rest was applied to a TLC plate. The plate was first developed in a polar solvent (chloroform/methanol/acetic acid/water (90:15:10:3 v/v/v) to half its height, dried, and then fully developed in heptane:diethylether:acetic acid (70:30:1 v/v/v). Phosphatidylcholine and free fatty acids were scraped from the plate and methylated by addition of methanol containing 2% sulfuric acid at 90 ˚C for 30 min. The methyl esters were extracted in hexane and analyzed using RP-TLC or argentation-TLC as described for the desaturase assays. The upper (aqueous) phase of the reaction mixture extraction, that contained the acyl-CoAs, was hydrolyzed by addition of an equal volume of 2 M potassium hydroxide in methanol/water (1:4 v/v) and incubated for 20 min at 90 ˚C. Hydrolyzed fatty acids were extracted by addition of 3 M HCl (0.7 ml), methanol (1.4 ml) and chloroform (1.9 ml). The chloroform phase was recovered, dried under N_2_(g) and fatty acids were methylated and methyl esters extracted. An aliquot of the hexane phase was analyzed by scintillation counting and the remainder was separated by RP-TLC or argentation-TLC.

## Results

### *In Vivo* Feeding of Precursor Fatty Acids in Yeast Strains Expressing Elongases and Desaturases in DHA Biosynthesis

To better define which fatty acids serve as substrates for the seven desaturases and three elongases that were introduced into canola, *in vivo* feeding studies were conducted to monitor enzymatic conversion of 14 potential fatty acid substrates (Table 2). For this purpose, the respective genes (Table 1) present in the OLA to DHA pathway shown in Fig. 1 were individually expressed in yeast. The transformed cells were fed with potential fatty acid substrates, lipids were then extracted from the cells and analyzed for their fatty acid composition. When cultures were fed with 0.25 mM exogenous substrate at the beginning of induction the amounts of product and substrate and the desaturation and elongation percentages remained constant for approximately 48–92 h (results not shown).

**Table 2:** Potential substrate fatty acids were presented to growing yeast strains expressing individual desaturases or elongases

Substrate	Fatty acids	Product fatty acids									
		d12Des(*Ps*)	d6Des(*Ot*)	d6Elo(*Pp*)	d6Elo(*Tp*)	d5Des(*Tc*)	o3Des(*Pi* **)**	o3Des(*Pir*)	d5Elo(*Ot*)	d4Des(*Pl*)	d4Des(*Tc*)
16:1n-7	PO	16:2n-4 (32.4 ± 0.6)	n.d.	n.d.	n.d.	n.d.	n.d.	n.d.	n.d.	n.d.	n.d.
18:1n-9	OLA	18:2n-6 (57.0 ± 0.5)	18:2n-9 (12.3 ± 0.3)	n.d.	n.d.	n.d.	n.d.	n.d.	n.d.	n.d.	n.d.
18:2n-6	LNA	n.d.	18:3n-6 (68.8 ± 1.9)	20:2n-6 (20.9 ± 1.2)	20:2n-6 (2.2 ± 0.2)	n.d.	18:3n-3 (5.5 ± 0.8)	18:3n-3 (6.3 ± 0.3)	20:2n-6 (1.6 ± 0.2)	n.d.	n.d.
18:3n-3	ALA	n.d.	18:4n-3 (71.0 ± 0.7)	20:3n-3 (31.2 ± 0.2)	20:3n-3 (4.6 ± 0.4)	n.d.	n.d.	n.d.	20:3n-3 (15.4 ± 2.1)	n.d.	n.d.
18:3n-6	GLA	n.d.	n.d.	20:3n-6 (80.2 ± 0.8)	20:3n-6 (70.1 ± 1.4)	18:4n-3 (0.2 ± 0.1)	18:4n-3 (7.8 ± 0.5)	18:4n-3 (2.7 ± 0.1)	20:3n-6 (4.9 ± 0.2)	n.d.	n.d.
18:4n-3	SDA	n.d.	n.d.	20:4n-3 (85.7 ± 1.2)	20:4n-3 (85.6 ± 0.3)	n.d.	n.d.	n.d.	20:4n-3 (25.5 ± 1.7)	n.d.	n.d.
20:3n-6	DGLA	n.d.	n.d.	n.d.	n.d.	20:4n-6 (58.0 ± 2.0)	20:4n-3 (39.7 ± 2.4)	20:4n-3 (31.6 ± 3.4)	22:3n-6 (8.7 ± 1.1)	n.d.	20:4n-6 (1.2 ± 0.1)
20:4n-3	ETA	n.d.	20:5n-3 (2.9 ± 0.2)	n.d.	n.d.	20:5n-3 (86.2 ± 1.0)	n.d.	n.d.	22:4n-3 (80.2 ± 2.5)	n.d.	20:5n-3 (3.3 ± 0.2)
20:4n-6	ARA	n.d.	n.d.	22:4n-6 (2.4 ± 0.1)	n.d.	n.d.	20:5n-3 (40.0 ± 3.4)	20:5n-3 (45.4 ± 2.4)	22:4n-6 (61.9 ± 5.2)	n.d.	n.d.
20:5n-3	EPA	n.d.	n.d.	22:5n-3 (5.2 ± 0.3)	n.d.	n.d.	n.d.	n.d.	22:5n-3 (90.8 ± 1.0)	n.d.	n.d.
22:4n-6	DTA	n.d.	n.d.	n.d.	n.d.	n.d.	22:5n-3 (17.9 ± 0.4)	22:5n-3 (25.6 ± 1.2)	n.d.	22:5n-6 (44.3 ± 4.8)	22:5n-6 (17.3 ± 0.2)
22:5n-3	DPAn-3	n.d.	n.d.	n.d.	n.d.	n.d.	n.d.	n.d.	24:5n-3 (16.6 ± 2.4)	22:6n-3 (43.8 ± 3.8)	22:6n-3 (20.7 ± 1.4)
22:5n-6	DPAn-6	n.d.	n.d.	n.d.	n.d.	n.d.	22:6n-3 (4.8 ± 0.8)	22:6n-3 (6.9 ± 0.9)	n.d.	n.d.	n.d.
22:6n-3	DHA	n.d.	n.d.	n.d.	n.d.	n.d.	n.d.	n.d.	24:6n-3 (12.8 ± 1.8)	n.d.	n.d.

For each feeding experiment fatty acid products were identified and percentage conversions including standard deviations are indicated in parentheses (). Control experiments using yeast strains carrying an empty vector did not convert the fatty acids listed below to any of the products in this table. Percentage conversion for each tested fatty acid was calculated as: product × 100/(substrate + product)

*n.d.* conversion to a product was not detected

The fatty acids tested included all substrates in the pathway outlined in Fig. 1, as well as known fatty acids that could be produced through the activity of these enzymes, such as ALA and SDA [12, 24–29]. The results presented in Table 2 show that the introduced enzymes efficiently catalyzed the expected reactions, as indicated by conversion of precursor fatty acids to predicted products (refer to Fig. 1 for expected reactions). By using the codon-optimized versions of these genes and including all fatty acids in the pathway, rather than just the known substrates, we were able to generate data that aids in the prediction of fatty acids that might be produced in canola plants with the introduced enzymes. The desaturases were capable of discriminating against a wide range of fatty acids although with some exceptions, including: (a) the d6Des(*Ot*) appears to desaturate the delta-5 position of ETA resulting in EPA, and (b) the d4Des(*Tc*) catalyzes a delta-5 desaturation of both DGLA and ETA synthesizing ARA and EPA, respectively. These are minor side-reactions of the respective enzymes resulting in fatty acids that are accepted as substrates by other enzymes included in this pathway. On the other hand, the elongases generally accepted a broader range of fatty acid substrates. While the two delta-6 elongases showed similar conversion percentages for the main substrates GLA and SDA, d6Elo(*Pp*) showed higher levels of activity on LNA and ALA. Nonetheless, activity was highest with GLA and SDA, suggesting that these fatty acids would be the preferred substrate of the enzyme. The results of the *in vivo* feeding experiments suggest that DHA synthesis from OLA occurs via a network of reactions (Supplemental Fig. 1), rather than a simple linear pathway as depicted in Fig. 1.

### *In Vitro* Yeast Assays of Elongases Involved in the Pathway from OLA to DHA

After confirming that each of the introduced proteins listed in Table 1 can be expressed *in vivo* as a functionally active protein in yeast cells, membranes were prepared from yeast strains expressing individual desaturases or elongases to demonstrate *in vitro* enzymatic activity. Assays utilized a [^14^C]substrate that could be converted to a [^14^C]fatty acid product that was isolated as a derivatized methyl ester. [^14^C]Fatty acid methyl ester (FAME) enzymatic products were detected and quantified using a combination of TLC and electronic autoradiography.

**Fig. 2:**
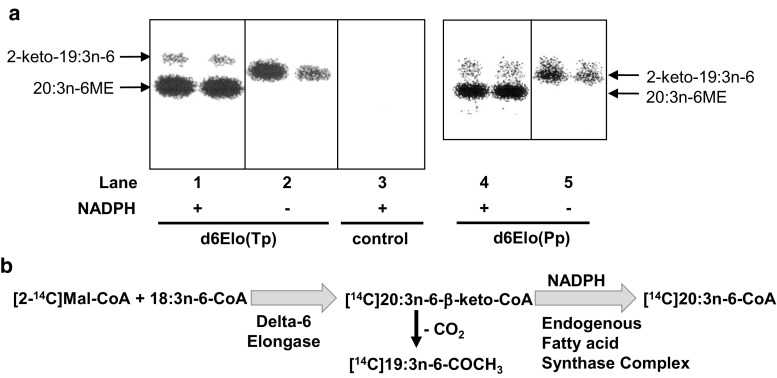
Demonstration of delta-6 elongase activities from *Thalassiosira pseudonana* (d6Elo(*Tp*)),* lanes 1* and* 2* in panel **a**, and *Physcomitrella patens* (d6Elo(*Pp*)),* lanes 4* and* 5* in panel **a**, using membrane preparations from yeast cells heterologously expressing these enzymes. Duplicate reactions are presented in each lane. Elongase assays included [^14^C]malonyl-CoA and 18:3n-6-acyl-CoA co-substrates in the presence (*lanes 1* and* 4*) or absence (*lanes 2* and* 5*) of NADPH. Depicted are autoradiographic images of TLC plates showing separated [^14^C]methyl esters (ME) prepared from the total lipids extracted from the enzymatic assays. The control assay, shown in* lane 3*, was performed with a membrane fraction isolated from a yeast strain harboring an empty vector and included NADPH. panel **b** shows the proposed reaction catalyzed by these delta-6 elongases

In the presence of NADPH, 18:3n-6-CoA, and [2-^14^C]malonyl-CoA, membranes isolated from yeast strains expressing either delta-6 elongase were capable of synthesizing a [^14^C]fatty acid (FA) that after methylation co-migrates with an authentic standard of 20:3n-6-methyl ester (ME) (Fig. 2, panel a, lanes 1 and 4). The [^14^C]20:3n-6FA is not produced by membranes that do not express a delta-6 elongase, as shown in Fig. 2, panel a, lane 3 or in the absence of 18:3n-6-CoA (data not shown). In the absence of NADPH membranes expressing the delta-6 elongases do not generate a reduced [^14^C]20:3n-6FA, but instead produce a faster migrating radioactive molecular species. This compound was previously characterized as a 2-ketone, [^14^C]2-keto-19:3n-6 (Fig. 2, panel a, lanes 2 and 5) produced by decarboxylation of the 3-keto condensing product [30, 31]. The decarboxylation occurs spontaneously during the assay (unpublished observation). The 2-ketone is also present in smaller amounts in the FAME fraction isolated from the reaction containing NADPH (Fig. 2, panel a, lanes 1 and 4) and results from an incomplete fatty acid synthesis cycle. Together these data support the reaction outlined in Fig. 2, panel b, in which the delta-6 elongases catalyze the transfer of two carbons from [^14^C]malonyl-CoA to 18:3n-6-CoA generating [^14^C]20:3n-6-beta-keto-CoA, which in the presence of NADPH, is reduced and ultimately converted to 20:3n-6-CoA by the endogenous yeast elongation complex enzymes [32] leading to the observed [^14^C]20:3n-6ME.

**Fig. 3:**
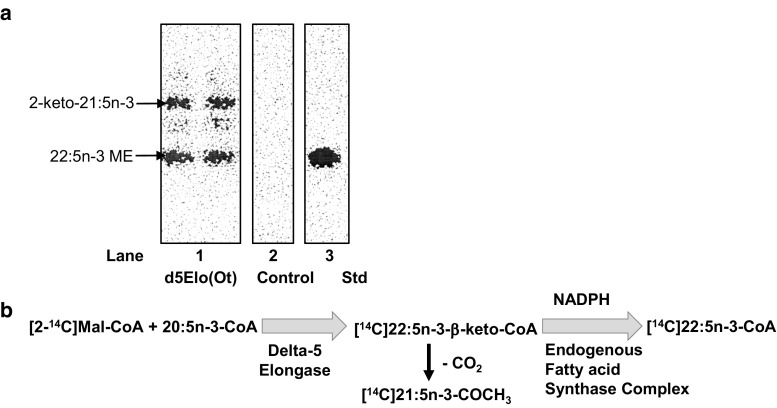
Demonstration of delta-5 elongase activity from *Ostreococcus tauri* (d5Elo(*Ot*)),* lane 1* in panel **a**, using membranes prepared from yeast cells heterologously expressing this enzyme. Duplicate reactions are presented in each lane. The elongase assay included the co-substrates [^14^C]malonyl-CoA and 20:5n-3-acyl-CoA in the presence of NADPH. Depicted are autoradiographic images of TLC plates showing separated [^14^C]methyl esters (ME) prepared from the total lipids extracted from the enzymatic assays. The control assay, shown in* lane 2* in panel **a**, was performed with a membrane fraction isolated from a yeast strain harboring an empty vector and included [^14^C]malonyl-CoA, 20:5n-3-acyl-CoA, and NADPH. An authentic [^14^C]methyl ester standard of 22:5n-3 is presented in* lane 3*, panel **a** for reference. Panel **b** shows the proposed reaction catalyzed by the delta-5 elongase from *Ostreococcus tauri*

Membranes isolated from yeast strains expressing the delta-5 elongase from *Ostreococcus tauri* (d5Elo(*Ot*)), when incubated with NADPH, 20:5n-3-CoA, and [2-^14^C]malonyl-CoA, generated a [^14^C]FA that after methylation co-migrated with an authentic standard of 22:5n-3ME (Fig. 3, panel a, lanes 1 and 3). The [^14^C]22:5n-3FA is not produced by membranes that do not express the d5Elo(*Ot*), as shown in Fig. 3, panel a, lane 2 or in the absence of 20:5n-3-CoA or [2-^14^C]malonyl-CoA (data not shown). As described for the delta-6 elongases, in the absence of NADPH, an incomplete fatty acid reduction cycle leads to the production of a faster migrating 2-ketone, [^14^C]2-keto-21:5n-3 compound (data not shown), which is also observed in the FAME isolated from the reaction containing NADPH (Fig. 3, panel a, lane 1). Together these data support the reaction outlined in Fig. 3, panel b in which the d5Elo(*Ot*) catalyzes the transfer of two carbons from [^14^C]malonyl-CoA to 20:5n-3-CoA generating [^14^C]22:5n-3-beta-keto-CoA, which in the presence of NADPH can be converted to 22:5n-3-CoA by the endogenous yeast elongation complex enzymes [32] leading to the observed [^14^C]22:5n-3ME.

### *In Vitro* Yeast Assays of Desaturases Involved in the Pathway from OLA to DHA

Fatty acid desaturases catalyze the abstraction of two hydrogen atoms from the hydrocarbon chain of a fatty acid to form a double bond in an unsaturated fatty acid and can be classified according to the backbone that their substrate is connected to: an acyl-CoA, an acyl-ACP (ACP, acyl carrier protein), or an acyl-lipid [33]. These enzymes utilize an electron donor in the reduction of the non-heme iron of the desaturase which may be a free cytochrome b_5_ protein or a cytochrome *b*_5_-like domain fused to the *N*-terminus of the desaturase [34]. In the presence of NADH the electron donor can be regenerated. Desaturases that accept acyl-ACP substrates are soluble enzymes [35]. The desaturases presented in Fig. 1 are predicted to have transmembrane spanning helices, similar to the crystal structures of membrane bound fatty acid desaturases [36, 37] and are therefore believed to utilize either an acyl-CoA or lipid-linked substrate. We first demonstrated desaturase activity using potential [^14^C]fatty acid substrates that were presented as a CoA ester that may (or may not) be converted to a phospholipid by endogenous acyl-CoA:lysophosphatidylcholine acyltransferase (LPCAT) and lyso-lipids present in the membranes that were prepared to test the desaturases. We then developed a method that provided stronger control over the backbone that contains the [^14^C]fatty acid.

**Fig. 4:**
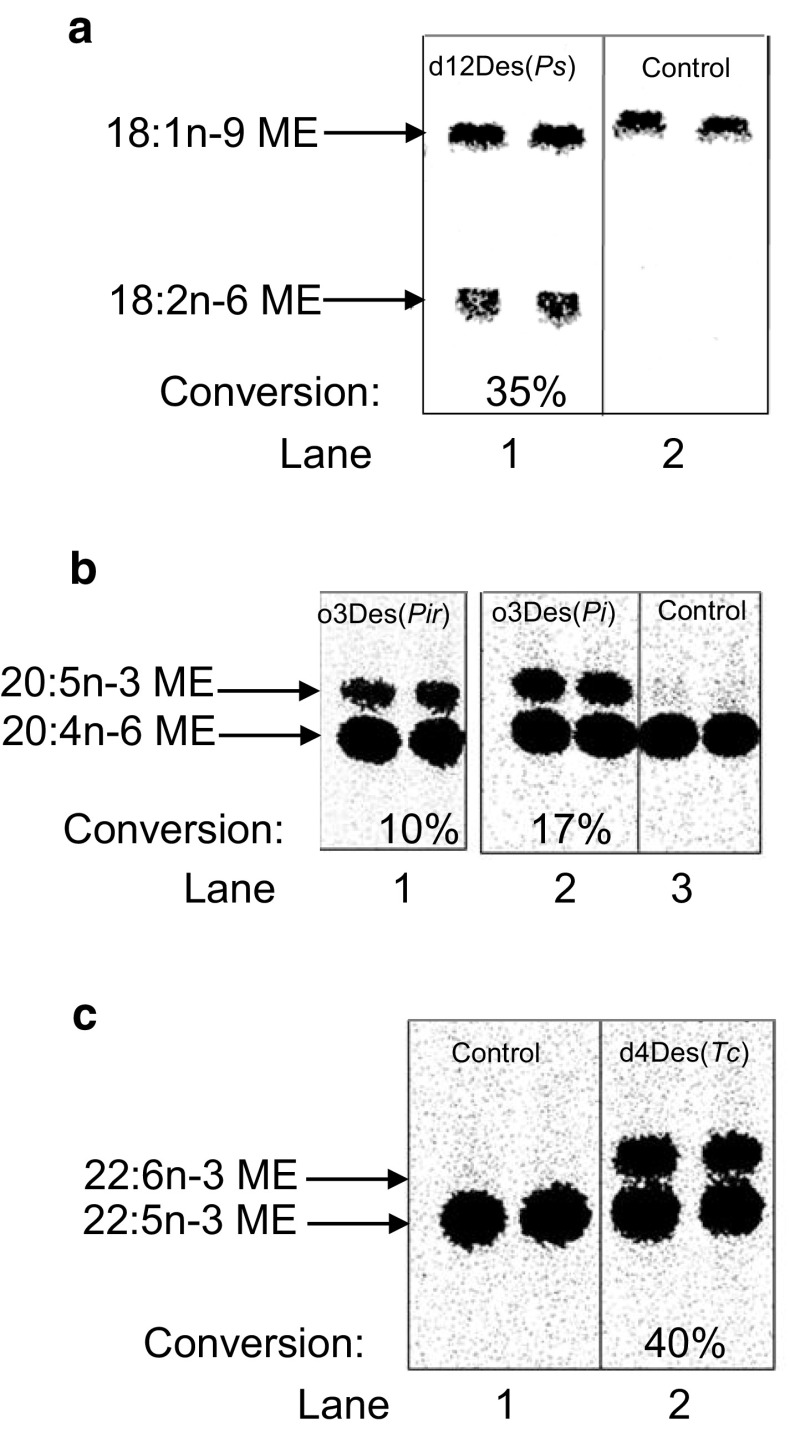
Demonstration of *in vitro* desaturase activities of delta-12 desaturase from *Phytophthora sojae* (d12Des(*Ps*)),* lane 1* in panel **a,** omega-3 desaturases from *Pythium irregulare* (o3Des(*Pir*)),* lane 1* in panel **b**, and *Phytophthora infestans* (o3Des(*Pi*)),* lane 2* in panel **b**, and delta-4 desaturase from *Thraustochytrium* sp. (d4Des(*Tc*)),* lane 2* in panel **c**. Duplicate reactions are presented in each* lane*. The activities were measured in membrane preparations of yeast cells heterologously expressing these enzymes. Control assays were performed with membrane fractions isolated from yeast strains harboring an empty vector (Control),* lane 2* in panel **a**,* lane 3* in panel **b** and* lane 1* in panel **c**. The figure depicts autoradiographic images of TLC plates of separated [^14^C]methyl esters (ME) prepared from the total lipids extracted from assays containing an [^14^C]acyl-CoA(d12Des(*Ps*), 18:1n-9; o3Des’s, 20:4n-6; and d4Des(*Tc*), 22:5n-3) and NADH. The conversion of [^14^C]substrate-fatty acid to [^14^C]product-fatty acid is shown for the desaturases as an average of the conversion in duplicate assays (% conversion). Lysophosphatidylcholine (lysoPtdCho) was added to each of the assays shown in this figure. Corresponding incubations without the addition of lysoPtdCho gave the following substrate conversions to product: d12Des(*Ps*), 6%; o3Des(*Pir*), 6%; o3Des(*Pi*), 8%; and d4Des(*Tc*), 20%

As shown in Fig. 4, membranes isolated from yeast strains expressing the delta-12 desaturase (panel a), both omega-3 desaturases (panel b) and the delta-4 desaturase from *Thraustrochytrium* sp. (panel c) are all capable of desaturating fatty acid substrates presented as CoA esters. All of these reactions required NADH (data not shown) and were not catalyzed by endogenous enzymes present in membranes prepared from yeast containing a control vector. However, in each of these examples, the desaturation reaction was stimulated by the addition of 16:0-lysophosphatidylcholine (lysoPtdCho) (see Fig. 4 legend).

**Fig. 5:**
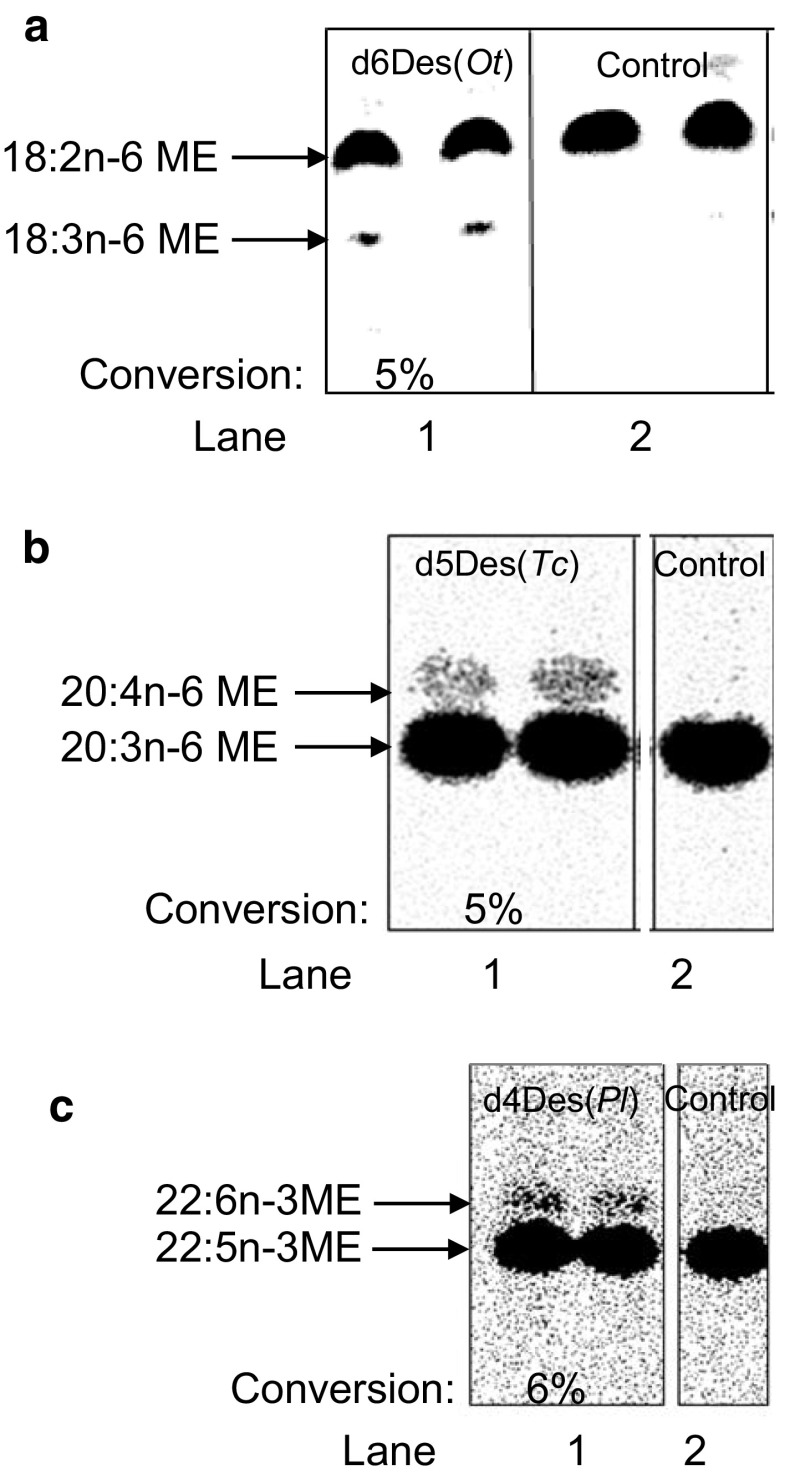
Demonstration of *in vitro* desaturase activities of delta-6 desaturase from *Ostreococcus tauri* (d6Des(*Ot*)),* lane 1* panel **a**, delta-5 desaturase from *Thraustochytrium* sp. (d5Des(*Tc*)),* lane 1* panel **b**, and delta-4 desaturase from *Pavlova lutheri* (d4Des(*Pl*)),* lane 1* panel **c**, in membrane preparations of yeast cells heterologously expressing these enzymes. Duplicate reactions are presented in each* lane*. Control assays were performed with membrane fractions isolated from yeast strains harboring an empty vector (Control),* lane 2* in panels **a**–**c**. The figure depicts autoradiographic images of TLC plates of separated [^14^C]methyl esters (ME) prepared from the total lipids extracted from the assays containing an [^14^C]acyl-CoA (d6Des(*Ot*), 18:2n-6; d5Des(*Tc*), 20:3n-6; and d4Des(*Pl*), 22:5n-3) and NADH. The conversion of [^14^C]substrate-fatty acid to [^14^C]product-fatty acid is shown for the desaturases as an average of the conversion in duplicate assays (% conversion). All enzyme assays shown in this figure were performed without the addition of lysoPtdCho. Corresponding incubations when lysoPtdCho was added to the assay resulted in the following substrate to product conversions: d6Des(*Ot*), 3%; d5Des(*Tc*), 3%; d4Des(*Pl*), 2%

As shown in Fig. 5, membranes isolated from yeast strains expressing the delta-6 desaturase (panel a), the delta-5 desaturases (panel b) and the delta-4 desaturase from *Pavlova lutheri* (panel c) are all capable of desaturating fatty acid substrates presented as CoA esters, but significantly less efficiently than the desaturases presented in Fig. 4. All of these reactions also required NADH (data not shown) and were not catalyzed by endogenous enzymes present in membranes prepared from yeast containing a control vector. However, unlike the data presented in Fig. 4, none of these desaturation reactions were stimulated by the addition of lysoPtdCho (see Fig. 5 legend), and in fact the reactions could be slightly inhibited by the addition of lysoPtdCho.

### Differentiation Between Desaturation of Fatty Acids Covalently Bound to PtdCho Or CoA

As shown for the enzymes presented in Fig. 4, including lysoPtdCho in the reaction dramatically stimulated fatty acid desaturation. A possible explanation is that during the reaction the [^14^C]fatty acid was transferred from CoA to the added lysoPtdCho by endogenous acyltransferases that are present in the membranes, thus providing (additional) PtdCho containing the [^14^C]fatty acid, which is the substrate for the desaturase. These same acyltransferases could also transfer the [^14^C]fatty acid from CoA to endogenous lipids by acyl exchange [38] or by acylation of endogenous lyso-lipids, which are also present in low levels in membranes, thereby providing an acyl-lipid substrate for the desaturases explaining the desaturation that is observed without including additional lysoPtdCho.

In order to distinguish between desaturation of acyl-CoA and lipid-linked fatty acids, we developed assay conditions that allowed better control over the carrier that contains the [^14^C]fatty acid substrate. The initial substrate added in the assays was a [^14^C]acyl-CoA and reactions were terminated with a biphasic methanol/chloroform extraction [23] in the presence of bovine serum albumin. In this extraction, acyl-CoAs are nearly exclusively recovered in the upper methanol/water phase or in the denatured protein pad at the interface, whereas phospholipids like phosphatidylcholine are exclusively found in the lower chloroform phase. However, simply analyzing the distribution of [^14^C]acyl substrate and [^14^C]product between the upper phase/protein pad and lower phase in such extractions is not sufficient to distinguish between desaturation of lipid-linked and acyl-CoA substrates. The desaturated product attached to CoA could, for example, be rapidly transferred to complex lipids, giving a false impression of desaturation occurring on a lipid-linked substrate. Yeast membranes also contain high levels of acyl-CoA thioesterase activity [39] that rapidly breaks down acyl-CoA substrates to free fatty acids, making it difficult to recover desaturated acyl-CoA enzymatic products.

To assay more specifically for acyl-CoA linked desaturation, we included a pre-incubation with 20:1n-9-CoA and DTNB for 10 min, before adding the [^14^C]acyl-CoA substrate and NADH co-reductant. The 20:1n-9-CoA addition serves two functions. First, it is used by endogenous lysophospholipid acyltransferase (ALE1) [40–43] to acylate endogenous lysophospholipids present in the membranes, thereby preventing the [^14^C]acyl-CoA from being incorporated into a phospholipid. Secondly, 20:1n-9-CoA reduces the extent to which the [^14^C]acyl-CoA is hydrolyzed to a free fatty acid, by non-specific acyl-CoA thioesterases, by increasing the total acyl-CoA substrate pool. DTNB prevents the reverse reaction of lysophospholipid acyltransferases, thereby prohibiting [^14^C]acyl groups from entering phospholipids by this route [38].

**Fig. 6:**
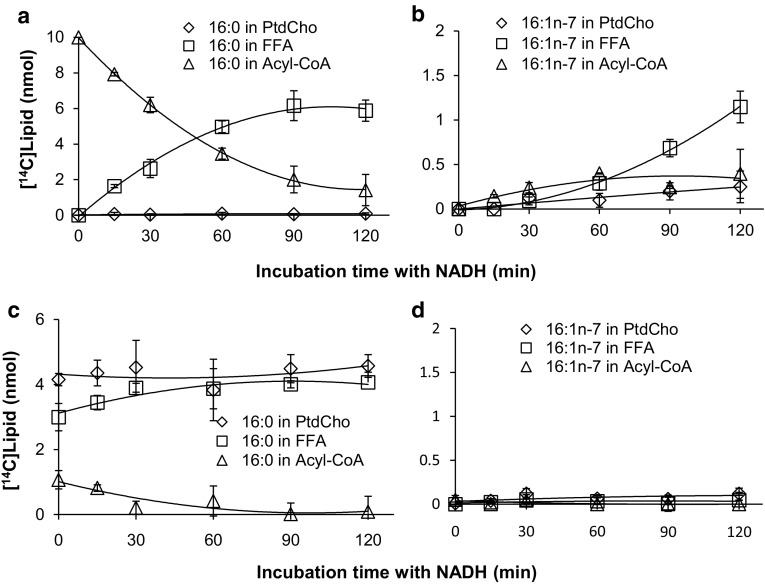
Substrate utilization of the *S. cerevisiae* delta-9 desaturase (d9Des(*Sc*)). A membrane preparation from yeast cells overexpressing the endogenous delta-9 desaturase was tested for its ability to use acyl-CoA (panels **a**, **b**) and phosphatidylcholine-linked (panels **c**, **d**) fatty acid substrates. In panels **a** showing substrate depletion, and **b** showing product accumulation, the membrane preparations were pre-incubated with DTNB and 20:1n-9-CoA for 10 min and the desaturase reaction was initiated by adding [^14^C]16:0-CoA substrate and NADH (time point zero in the graph) and further incubated for times as shown in the figure. In panels **c** showing substrate depletion, and **d** showing product accumulation, the membrane preparations were pre-incubated with [^14^C]16:0-CoA and lysophosphatidylcholine for 15 min and the desaturase reaction was initiated by addition of NADH and further incubated for time periods as shown in the figure. The results presented are from triplicate assays with the standard deviation as* error bars*. In each panel [^14^C]substrates and [^14^C]products were followed as: free fatty acids (*unfilled squares*, FFA); phosphatidylcholine (*unfilled diamonds*, PtdCho); and acyl-CoA (*unfilled triangles*)

**Fig. 7:**
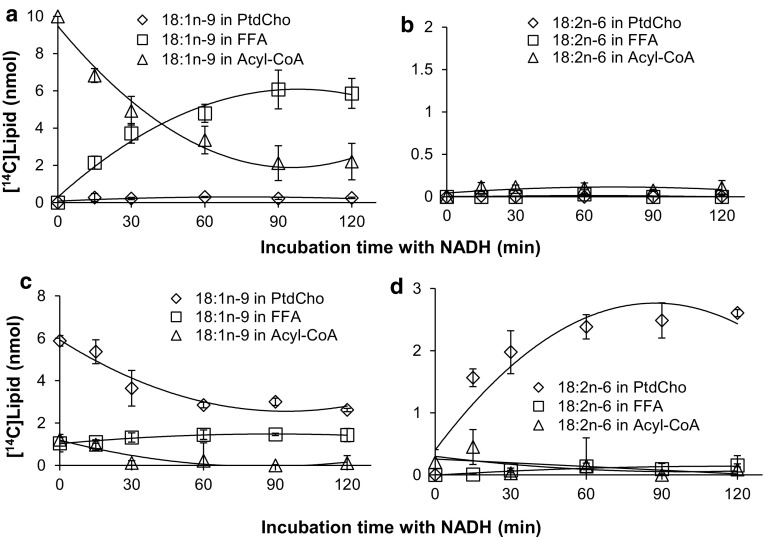
Substrate utilization of a *Phytophthora sojae* delta-12 desaturase. A membrane preparation from yeast cells expressing *Phytophthora sojae* delta-12 desaturase was tested for CoA linked acyl-desaturation (panels **a**, **b**) and phosphatidylcholine linked acyl-desaturation (panels **c**, **d**). In panels **a**, showing substrate depletion, and **b** showing product accumulation, the membrane preparations were pre-incubated with DTNB and 20:1n-9-CoA for 10 min and the desaturase reaction was initiated by adding [^14^C]18:1n-9-CoA substrate and NADH (time point zero in the graph) and further incubated for times as shown in the figure. In panels **c** showing substrate depletion, and **d** showing product accumulation, the membrane preparations were pre-incubated with [^14^C]18:1n-9-CoA and lysophosphatidylcholine for 15 min and the desaturase reaction was initiated by addition of NADH and further incubated for times as shown in the figure. The results presented are from triplicate assays with the standard deviation as* error bars*. In each panel [^14^C]substrates and [^14^C]products were followed as: free fatty acids (*unfilled squares*, FFA); phosphatidylcholine (*unfilled diamonds*, PtdCho); and acyl-CoA (*unfilled triangles*)

**Fig. 8:**
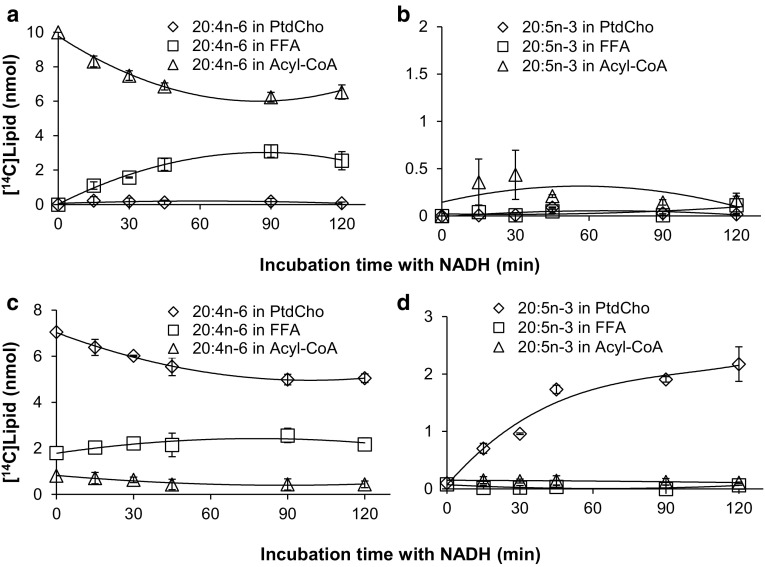
Substrate utilization of a *Phytophthora infestans* omega-3 desaturase. A membrane preparation from yeast cells expressing *Phytophthora infestans* omega-3 desaturase was tested for CoA linked acyl-desaturation (panels **a**, **b**) and phosphatidylcholine linked acyl-desaturation (panels **c**, **d**). In panels **a**, showing substrate depletion, and **b** showing product accumulation, the membrane preparations were pre-incubated with DTNB and 20:1n-9-CoA for 10 min and the desaturase reaction was initiated by adding [^14^C]20:4n-6-CoA substrate and NADH (time point zero in the graph) and further incubated for times as shown in the figure. In panels **c** showing substrate depletion, and **d** showing product accumulation, the membrane preparations were pre-incubated with [^14^C]20:4n-6-CoA and lysophosphatidylcholine for 15 min and the desaturase reaction was initiated by addition of NADH and further incubated for times as shown in the figure. The results presented are from triplicate assays with the standard deviation as* error bars*. In each panel [^14^C]substrates and [^14^C]products were followed as: free fatty acids (*unfilled squares*, FFA); phosphatidylcholine (*unfilled diamonds*, PtdCho); and acyl-CoA (*unfilled triangles*)

**Fig. 9:**
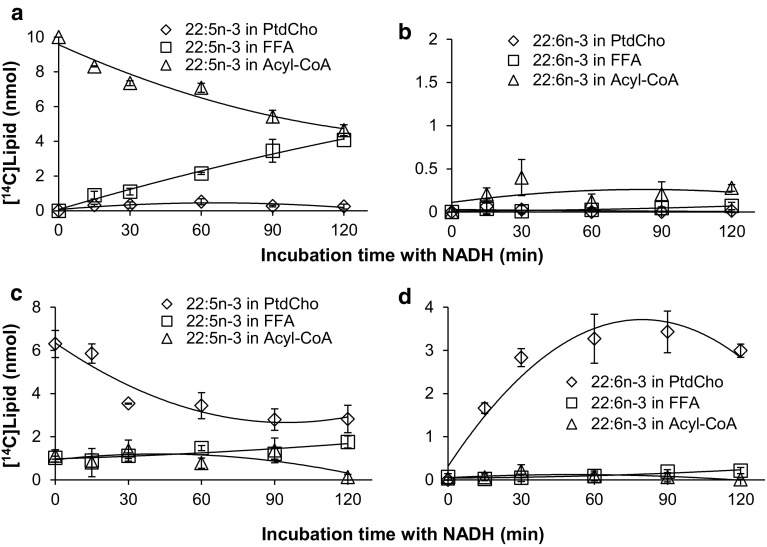
Substrate utilization of a *Thraustrochytrium* delta-4 desaturase. A membrane preparation from yeast cells expressing *Thraustrochytrium* delta-4 desaturase was tested for CoA linked acyl-desaturation (panels **a**, **b**) and phosphatidylcholine linked acyl-desaturation (panels **c**, **d**). In panels **a** showing substrate depletion, and **b** showing product accumulation, the membrane preparations were preincubated with DTNB and 20:1n-9-CoA for 10 min and the desaturase reaction was initiated by adding [^14^C]22:5n-3-CoA substrate and NADH (time point zero) and further incubation for times as shown in the figure. In panels **c** showing substrate depletion, and **d** showing product accumulation, the membrane preparations were pre-incubated with [^14^C]22:5n-3-CoA and lysophosphatidylcholine for 15 min and the desaturase reaction was initiated by addition of NADH and further incubated for times as shown in the figure. The results presented are from triplicate assays with the standard deviation as* error bars*. In each panel [^14^C]substrates and [^14^C]products were followed as: free fatty acids (*unfilled squares*, FFA); phosphatidylcholine (*unfilled diamonds*, PtdCho); and acyl-CoA (*unfilled triangles*)

To test for desaturation of lipid-linked fatty acids we specifically looked at phosphatidylcholine (PtdCho) linked fatty acids. In this case, the membranes were pre-incubated for 15 min with the [^14^C]acyl-CoA and lysophosphatidylcholine. The yeast lysophospholipid acyltransferase (ALE1) enzyme efficiently transferred the added [^14^C]acyl-CoA and after 15 min nearly all the [^14^C]acyl-CoA had been metabolized into phosphatidylcholine or hydrolyzed to free fatty acids (see Figs. 6c, 7c, 8c, 9c). The latter product was presumably generated by endogenous thioesterases that are also present in the membranes. After 15 min, NADH was added to initiate desaturation, and the distribution of radioactivity in the acyl groups was determined at different time intervals.

The integral membrane delta-9 desaturase, in a reaction that uses molecular oxygen, NADH-cytochrome *b*_5_ reductase and cytochrome *b*_5_, introduces a double bond between carbons 9 and 10 of stearoyl-CoA, as well as other fatty acids covalently bound to CoA [36, 37, 44]. We therefore used the *S. cerevisiae* delta-9 desaturase (OLE1 [21], here referred to as d9Des(*Sc*)) as a control to demonstrate our method and these results are shown in Fig. 6. Membrane preparations from yeast cells overexpressing its own delta-9 desaturase were first assayed for desaturation of acyl-CoA substrates. Figure 6, panel a depicts the fate of [^14^C]16:0 after addition of [^14^C]16:0-CoA in the presence of NADH. In this experiment the membranes were pre-incubated with 20:1n-9-CoA and DTNB for 10 min, and then the NADH and [^14^C]16:0-CoA were added (zero time point). A significant amount of [^14^C]16:0 existed as an acyl-CoA throughout the 120 min period that was monitored (Fig. 6, panel a). However, 60% of the added radioactivity was recovered as a free fatty acid after 90 min, indicating that thioesterase activity on the substrate was only partially averted. The [^14^C]16:0 found in PtdCho was minimal (0.4%). The desaturated [^14^C]16:1n-7 product was only found in the acyl-CoA fraction after a 15 min incubation with NADH and [^14^C]16:1 in this fraction peaked after 60 min of incubation (Fig. 6, panel b). [^14^C]16:1n-7 free fatty acid accumulated after 15 min and continued to increase throughout the time-course, containing about 12% of the added radioactivity after 120 min (Fig. 6, panel b). Despite the small amount of [^14^C]16:0 found in PtdCho, a significant amount of [^14^C]16:1n-7 accumulated in PtdCho throughout the time course and at the end of 120 min amounted to 4% of added [^14^C]radioactivity which was 10 times more than the [^14^C]16:0 found in this lipid. These results can most simply be interpreted as desaturation of 16:0-CoA along with thioesterases acting on the acyl-CoA pool to produce free 16:0 and 16:1n-7 fatty acids. However, it cannot be excluded that a certain amount of [^14^C]16:0 entered the PtdCho pool and was efficiently desaturated on this lipid. Alternatively, since LPCAT significantly prefers 16:1n-7-CoA as a substrate relative to 16:0-CoA [40–42], 16:1n-7 may have been efficiently transferred from CoA to PtdCho. To investigate whether the d9Des(*Sc*) uses PtdCho-linked substrates in addition to acyl-CoA substrates, the membrane fractions were pre-incubated with added lysoPtdCho for 15 min, and about 50% of the recovered radioactivity was in the form of [^14^C]16:0-PtdCho, 40% in free fatty acid and 10% in the acyl-CoA fraction (Fig. 6, panel c, time point zero). The desaturation reaction was then initiated by adding NADH. The radioactivity recovered in 16:0-CoA decreased from 10% to near zero after 60 min of incubation with NADH, whereas 16:0 free fatty acid increased from 40 to 50% of recovered radioactivity and 16:0-PtdCho remained more or less constant throughout the 120 min time course. The total recovery of [^14^C]16:1n-7 was 16 times less than that seen in assays measuring desaturation of acyl-CoAs and was recovered in PtdCho and free fatty acids after 120 min incubation (Fig. 6, panel d). This experiment clearly shows that desaturation of [^14^C]16:0 is extremely low when the substrate acyl group is captured in PtdCho and the small amount of [^14^C]16:1n-7 recovered could entirely be explained by desaturation of the remaining 10% of 16:0-CoA (present upon addition of NADH) and its efficient transfer into PtdCho. Thus desaturation of PtdCho-linked substrates can be excluded for the *S. cerevisiae* delta-9 desaturase.

To determine the acyl carrier of the substrate fatty acid for the delta-12 desaturase from the oomycete *Phytophthora sojae*, the omega-3 desaturase from the oomycete *Phytophthora infestans* and the delta-4 desaturase from the Protista *Thrastochytrium* sp. we performed a similar suite of experiments as described above using membrane fractions prepared from yeast strains expressing each of these enzymes. For each of these desaturases, the results regarding substrate utilization were in sharp contrast to the results observed for the yeast delta-9 desaturase. In the assays observing desaturation of acyl-CoAs, very little of the presented [^14^C]acyl-CoA entered PtdCho and the small amount of radioactive desaturation product that accumulated existed as a free fatty acid or an acyl-CoA with low or very low amounts as a PtdCho (Panels a and b in Figs. 7, 8, 9). In the assays following desaturation of PtdCho substrates about 90% of the [^14^C]acyl-CoA was consumed during the 15 min incubation with lysoPtdCho, with 70–76% of the recovered radioactivity residing in PtdCho (time point zero, panel c in Figs. 7, 8, 9). Upon addition of NADH, desaturated product accumulated in the fatty acids bound to PtdCho (panel d Figs. 7, 8, 9) with a corresponding decrease in [^14^C]substrate in that lipid (panel c Figs. 7, 8, 9). No or very low levels of desaturation products were found in the acyl-CoAs and free fatty acids. The levels of desaturated products attached to PtdCho were 10–20 times higher than the levels of total desaturation products found when assaying for desaturation of acyl-CoA substrates with the same enzyme. The results clearly show that all three of these desaturases use PtdCho linked acyl substrates. However a very minor level of acyl-CoA linked desaturation cannot be excluded since small amounts of desaturated products were found in the acyl-CoA fraction and in free fatty acids in the assays for acyl-CoA linked desaturation.

## Discussion

Generation of EPA and DHA in plant seed oil could provide a new and renewable commercial source for these health giving omega-3 fatty acids. Toward this goal we have previously shown that upon introduction of 10 genes, encoding for desaturases and elongases, canola (*B. napus*) is capable of producing a seed oil containing significant levels of EPA and DHA [18]. To better understand how endogenous seed-fatty acids are converted into EPA and DHA by the collective action of the enzymes introduced into canola seed we present yeast *in vivo* and *in vitro* activities of these three elongases and seven desaturases. We also developed a novel *in vitro* method to distinguish between desaturases that utilize fatty acid substrates covalently bound to CoA from phosphatidylcholine.

The *in vivo* feeding studies demonstrated that each desaturase and elongase is capable of recognizing multiple fatty acid substrates. In the context of the conversion of OLA to DHA, this means that each enzyme can catalyze multiple reactions in the pathway, and that each reaction may be catalyzed by multiple enzymes. Therefore, when the 10 enzymes are combined in a heterologous system, such as a plant seed, one might expect DHA to be synthesized via a network rather than a linear series of reactions. The ability to model such a network will provide a more complete understanding of the fatty acid profile obtained when combining these 10 enzymes into a single cell or organism.

Desaturation of fatty acids can occur when the acyl group is linked to ACP as in the reaction catalyzed by the delta-9 desaturase in plant plastids [35], when the fatty acid is esterified to CoA as in the reaction catalyzed by the delta-9 stearoyl-CoA desaturase [36, 37, 44], or when it is covalently attached to a complex lipid, such as plant galactolipids [45] and phospholipids, notably phosphatidylcholine [46]. Only four membrane bound fatty acid desaturases have been purified [47–50], of which three have been shown to act on acyl-CoA. The fourth, a plastidial omega-6 desaturase, was in its solubilized form shown to accept a number of complex lipids as well as free fatty acid substrates [49, 51], although *in vivo* experiments suggest that monogalactosyldiacylglycerols (MGDG) and digalactosyldiacylglycerols (DGDG) are the sole substrates in plant cells [45].

In order to untangle the regulation of fatty acid and lipid metabolism in a cell, it is essential to know the substrates for the different enzymatic reactions involved. This is best accomplished using *in vitro* systems where pool sizes of different lipids and acyl exchange between different pools can be strictly controlled. Very few such *in vitro* systems for fatty acid desaturase reactions have been described, and they are all in plants [46, 52–55]. Thus, the evidence for the actual substrates for most acyl desaturases is still lacking, making the interpretation of overall fatty acid and lipid metabolism in cells expressing these enzymes somewhat speculative rather than based on scientific evidence. Understanding the biosynthetic pathway for production of EPA and DHA from oleic acid illustrates the importance of knowing the actual substrate to which the different acyl groups are attached when they are desaturated. That the elongation of C18 acyl groups to very long chain acyl groups (≥C20) uses acyl-CoA as substrate is firmly established [32]. Two elongation steps and five desaturation steps are needed for the conversion of oleic acid into DHA. If the desaturation step preceding the elongation requires lipid linked substrates, it necessitates that the desaturated acyl group is transferred from that lipid into the acyl-CoA pool, whereas with an acyl-CoA linked desaturation, elongation can take place directly on the desaturated acyl-CoA product.

Using a novel experimental strategy that does not require purified proteins we show that the *S. cerevisiae* delta-9 desaturase (OLE1 or d9Des(*Sc*)), similar to its well characterized mammalian orthologues, utilizes acyl-CoA substrates. Membrane fractions prepared from yeast cells overexpressing d9Des(*Sc*) were tested in two different assays that were designed to dictate whether the substrate fatty acid [^14^C]16:0 was a CoA ester or bound to PtdCho. In the assay testing desaturation of acyl-CoAs, the 16:1 enzymatic product accumulated primarily in the acyl-CoA fraction and as free fatty acids. A very small amount of radioactivity entered PtdCho (4% of added radioactivity) but 90% of this radioactivity resided in 16:1n-9. These results could be interpreted as indicating that the major desaturation occurred on acyl-CoA but 16:0 entering PtdCho was also efficiently desaturated. However, when the d9Des(*Sc*) enzyme was assayed for desaturation of PtdCho substrates, the amount of 16:1n-9 in PtdCho was minute (1% of added radioactivity) even though this lipid contained 50% of the recovered radioactivity. This confirms that the yeast delta-9 desaturase uses acyl-CoA substrates with no activity towards acyl groups esterified in PtdCho. In the assays testing for acyl-CoA desaturation, about the same amount of [^14^C]16:1n-9 was found in PtdCho and the acyl-CoA fraction, despite that the latter fraction contained seven times more radioactivity, thus supporting previous reports that 16:1n-9 is significantly preferred over 16:0 by LPCAT [40–42]. Such experimental evidence might lead to erroneous conclusions about the true substrates of the desaturases if it was not complemented with the experiments specifically testing for desaturation of PtdCho substrates.

The assays of the delta-12 desaturase from the Oomycete *P. sojae*, the omega-3 desaturase from the Oomycete *P. infestans* and the delta-4 desaturase from the Protista *Thraustochytrium* show clearly that they utilize PtdCho-linked substrates and their ability to accept acyl-CoA substrates is very low. This is, as far as we know, the first time that lipid-linked fatty acid desaturation has convincingly been shown in eukaryotic kingdoms other than Plantae and confirms the theory that the dichotomy in substrate utilization for desaturation and elongation may limit DHA synthesis in plants [16]. Our strategy of using endogenous LPCAT activity to selectively acylate a lyso-lipid to *in situ* generate the desaturase substrate could potentially be further modified to use other lyso-lipids to form additional potential phospholipid substrates (e.g. phosphatidylethanolamine and phosphatidylserine).

Seven desaturase enzymes were assayed for total [^14^C]acyl desaturation from added [^14^C]acyl-CoA with and without addition of lysoPtdCho in the assay mixture. In these assays the total acyl groups in the assays were methylated and separated using TLC based on their degree of desaturation. Like the three desaturases that were further shown to prefer PtdCho-linked substrates, the omega-3 desaturase from *Pythium irregulare* had significantly higher activity when assayed with addition of lysoPtdCho than when lysoPtdCho was omitted. On the other hand, the delta-6 desaturase from *Ostreococcus tauri*, the delta-5 desaturase from *Thraustochytrium* sp. and the delta-4 desaturase from *Pavlova lutheri* had lower activity when lysoPtdCho was added, suggesting that these enzymes use an acyl-CoA substrate. However, the total conversion of added [^14^C]acyl groups was much lower for these three desaturases than the others. Due to the low amount of radioactive product formed, conclusive results could not be obtained for these enzymes in the assays discriminating between desaturation of acyl-CoA and PtdCho linked fatty acids. Indications that the delta-6 desaturase from *Ostreococcus tauri* utilizes acyl-CoA substrates has also previously been obtained from yeast based co-expression studies [24].

To date, reports of the production of the healthy omega-3 fatty acids EPA and DHA in plant seed oil have focused on introducing enzymes that synthesize and modify the fatty acid chain [12–15]. Networks employing discrete elongases and desaturases have led to the generation of significant levels of EPA and DHA [12–14], despite the fact that at least some of these desaturases use PtdCho-linked substrates, as shown in our work here. However, higher levels of EPA and DHA could be achieved by also introducing specialized acyltransferases that transfer the growing intermediates from PtdCho into the acyl-CoA pool and channel the end products to triacylglycerols [56]. Thus, the use of the assay methods developed in this work will aid in understanding the biochemical pathways of certain unsaturated fatty acids found in triacylglycerols in different organisms as well as guide in deciding strategies to produce oils with high amounts of these fatty acids in engineered seeds.

## Electronic supplementary material

Below is the link to the electronic supplementary material.

Supplementary material 1 (PDF 144 kb)

## Abbreviations

ALA: Alpha-linolenic acid
ARA: Arachidonic acid
BSA: Bovine serum albumin
DGLA: Dihomo-gamma-linolenic acid
DHA: Docosahexaenoic acid
EPA: Eicosapentaenoic acid
ETA: Eicosatetraenoic acid
FA: Fatty acid(s)
FAME: Fatty acid methyl ester(s)
GLA: Gamma-linolenic acid
LNA: Linoleic acid
lysoPtdCho: Lysophosphatidyl choline
ME: Methyl ester(s)
OLA: Oleic acid
PtdCho: Phosphatidylcholine
SDA: Stearidonic acid
TLC: Thin layer chromatography
