# Supplementary material for: Determination of Substrate Preferences for Desaturases and Elongases for Production of Docosahexaenoic Acid from Oleic Acid in Engineered Canola
Source: Lipids. 2017 Feb 14;52(3):207–22. doi: 10.1007/s11745-017-4235-4 (PMC5325871; doi:10.1007/s11745-017-4235-4)
Supplement: Supplementary file 1 — Supplementary material 1 (PDF 144 kb) [file 11745_2017_4235_MOESM1_ESM.pdf]

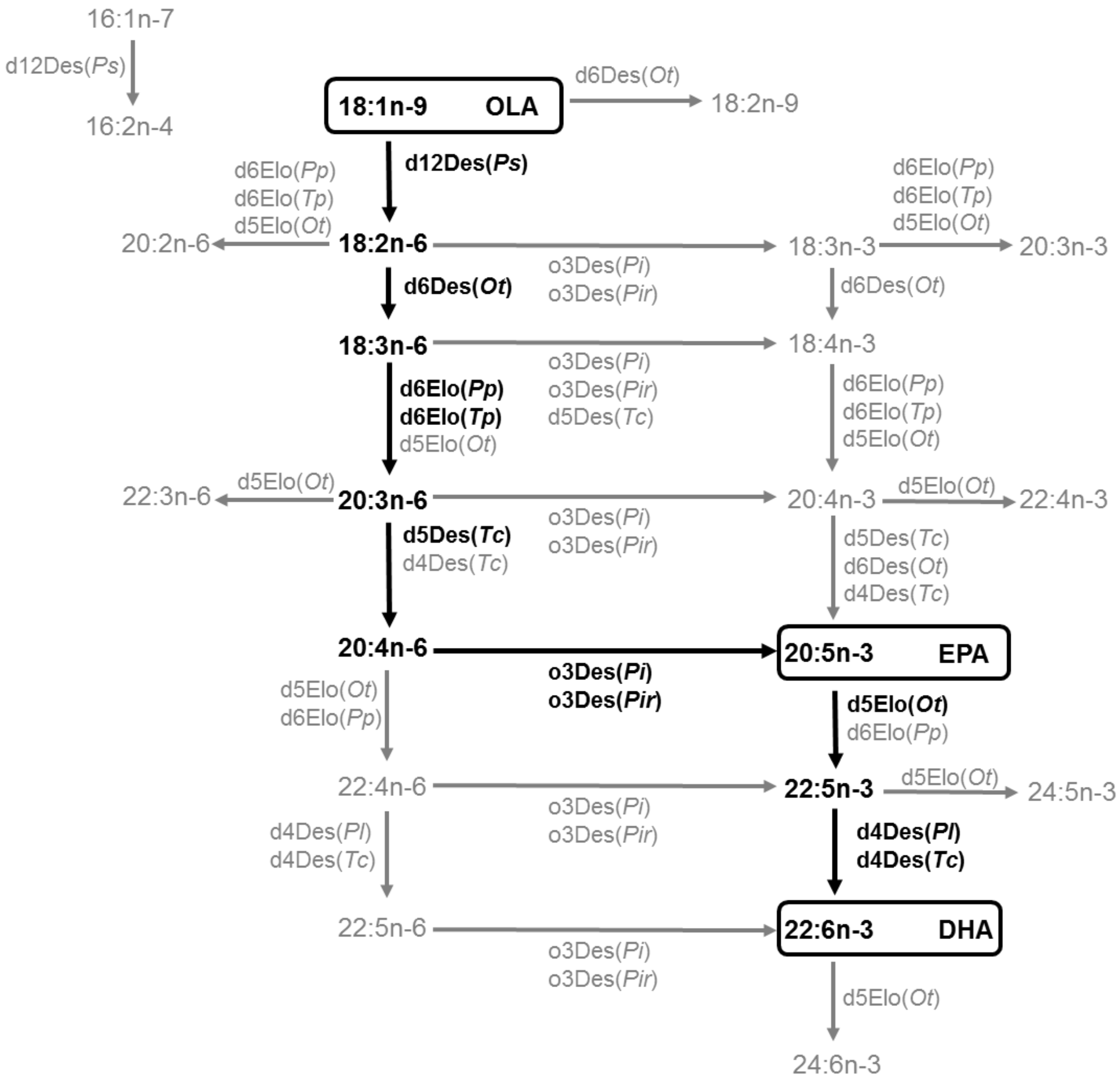

Supplemental Figure 1. Fatty acid specificity data from Table 2 mapped onto the pathway depicted in Figure 1. Enzymes capable of each reaction are listed next to the reaction arrows. The core pathway from Figure 1 is shown with black text, while additional reactions are shown with gray text. The seven desaturase enzymes (Des) and three elongase enzymes (Elo) form a network capable of synthesized DHA from OLA via many different routes. Acyl-carrier specificities are not taken into account in this depiction of the biosynthetic network.
